# Factors Associated with Avoiding Referrals by Dental Teleconsulting Sessions in Brazil

**DOI:** 10.3390/ijerph20065104

**Published:** 2023-03-14

**Authors:** Lígia C. Paixão, Mauro Henrique N. G. Abreu, Antônio P. Ribeiro-Sobrinho, Renata C. Martins

**Affiliations:** 1 School of Dentistry, Universidade Federal de Minas Gerais, Belo Horizonte 31270-901, MG, Brazil; 2 Department of Community and Preventive Dentistry, School of Dentistry, Universidade Federal de Minas Gerais, Belo Horizonte 31270-901, MG, Brazil; 3 Department of Restorative Dentistry, School of Dentistry, Universidade Federal de Minas Gerais, Belo Horizonte 31270-901, MG, Brazil

**Keywords:** telehealth, primary health care, referral and consultation, dentistry, COVID-19

## Abstract

This cross-sectional analytical study assessed the frequency of avoided referrals of primary care to other care levels by dental teleconsulting and its association with individual and contextual variables using a multilevel approach. It appraised asynchronous dental teleconsulting sessions from the secondary database of the Monitoring and Evaluation System of the Telehealth Results during 2020, during the COVID-19 pandemic. The outcome was “whether referral to secondary care was avoided”. Individual variables were related to teleconsulting and professionals that requested it: sex, dental specialty, and dentistry field. Contextual variables were related to each municipality that requested responses: Municipal Human Development Index, oral health teams (OHTs) in primary health care coverage, dental specialty centers coverage, illiteracy rate, Gini index, longevity, and per capita income. A descriptive analysis was made using the Statistical Package for the Social Sciences. Hierarchical Linear and Nonlinear Modeling software was used to perform multilevel analyses to assess the association of individual and contextual variables with avoiding patient referral to other care levels. Most teleconsulting sessions avoided patient referral to other care levels (65.1%). Contextual variables explained 44.23% of the variance in the outcome. Female dentists were more likely to avoid patient referrals than male dentists (OR = 1.74; CI = 0.99–3.44; *p* = 0.055). In addition, an increase of one percentage point in OHT/PHC coverage of municipalities increased the likelihood of avoiding patient referral by 1% (OR = 1.01; CI = 1.00–1.02; *p* = 0.02). Teleconsulting sessions efficiently avoided patient referral to other care levels. Both contextual and individual factors were associated with avoided referrals by teleconsulting sessions.

## 1. Introduction

The stimulated gatekeeping of primary health care (PHC) and regulation of referrals to specialized care is a global issue [1]. In Brazil, its continental dimension and heterogeneous health services coverage are challenges to achieving equity in health care [1]. The Brazilian PHC is the gateway to initial care and solving most population problems [2]. When a health condition cannot be treated in the PHC, it must be referred to specialized care services [3].

Despite implementing strategies to overcome these obstacles, the Brazilian Unified Health System (SUS) network in PHC and specialized care services, such as the dental specialty centers (DSCs), is quite diverse in structure, regional distribution, and financing [1,3]. It results in long-distance travel, long waiting lists [3], and hampered communication between PHC professionals and specialists [1].

Telehealth and teledentistry use technological resources for communication in health areas [4] and may support PHC and referral management, allowing remote communication between patients and professionals (teleconsultation) [5]. They also enable teletriage, telediagnosis, and monitoring of the progress of treatment outcomes (telemonitoring) [5]. The benefits for the health systems are a reduction in unnecessary referrals to specialized care, streamlined resources [6], and downsized waiting lists [7]. These initiatives became more critical during the COVID-19 pandemic in 2020 [8] since they could contribute to social distancing [9].

The Brazilian Ministry of Health implemented telehealth in 2006 to strengthen PHC and increase access to specialized health in the country, which is currently known as the Telehealth Brazil Networks Program [10]. Unlike other national initiatives, remote communication provided by Brazilian telehealth is not allowed directly between patients and health professionals [11]. A resolution from the Federal Council of Dentistry during the COVID-19 pandemic allowed telemonitoring and teleadvice to guide the best moment to attend to patients [12].

Teleconsulting is one of the telehealth program’s strategies and consists of remote communication between the PHC professional and a specialist in a specific area called a teleconsultant. They exchange information about clinical care, health promotion actions, and work processes by messaging or videoconferences synchronously or asynchronous text messages that must be answered within 72 h. Teleconsulting is offered by telehealth centers, mostly linked to federal universities, potentially covering all Brazilian PHC [10,13]. Teleconsulting sessions can streamline the resolution of demands compatible with PHC’s attributions [4]. They may contribute to the referral system flow, increase PHC effectiveness and access to services, and foster the continuing education of professionals [4,9,14,15,16]. 

It is crucial to evaluate the resoluteness of the telehealth program’s dental teleconsulting sessions to avoid unnecessary referrals and its associated factors to plan policies that tackle inequalities. Thus, this study aimed to assess the prevalence of patient referrals avoided by dental teleconsulting sessions and their association with individual and contextual variables using a multilevel approach. The null hypothesis was that the prevalence of avoiding patient referral to other care levels by teleconsulting sessions does not differ between municipalities and individual factors.

## 2. Materials and Methods

A cross-sectional analytical study that appraised asynchronous dental teleconsulting sessions from the secondary database of the Telehealth Brazil Networks Program in 2020 was carried out. This was a population study of the entire Brazilian territory covered by this program. Data collection used an integrative platform that contains information on telehealth centers in the telehealth program: the Monitoring and Evaluation System of the Telehealth Results [17]. Codes from the International Classification of Diseases (ICD-10) and the International Classification of Primary Care 2 (ICPC-2) applied to dentistry were used to filter data [18]. As inclusion criteria, only questions requested by dentists that had information about the referral and not of a patient were analyzed. The research ethics committee of the Federal University of Minas Gerais (*Universidade Federal de Minas Gerais*) approved the research under protocol CAAE 17400319.9.0000.5149.

The outcome was avoiding referral to other care levels after each teleconsulting session, dichotomized into yes/no. The covariables were structured into individual (level 1) and contextual levels (level 2). The variables for the individual level were gathered from the database of the Monitoring and Evaluation System of the Telehealth Results [18]. The variables for the contextual level were collected from the database of the PNUD [19] and the National Information System of Brazil’s Ministry of Health [20,21,22] (Table 1).

IBM Statistical Package for Social Sciences v. 22.0 (IBM SPSS Statistics for Windows, Armonk, NY, USA) was used to perform descriptive analysis, and Hierarchical Linear and Nonlinear Modeling software (HLM 6.08 statistical package) to perform a multilevel analysis was used to assess the association of individual and contextual variables with avoiding patient referral to other care levels. The analysis included 1,319 teleconsulting sessions (level 1) from 279 municipalities (level 2). Parameters were estimated using the restricted maximum likelihood method and predictive quasi-likelihood estimation. A multilevel logistic regression model was built. In the first stage, a null model estimated the primary partition of data variability between two levels, and then the individual and contextual characteristics were considered. The variance partition coefficient (VPC) was calculated to determine how much variance in the outcome stems from the municipalities’ differences. Each was individually incorporated into the model before being tested for the association between the outcome and the covariables of levels 1 and 2 using Student’s *t*-test (*p* < 0.05). Because the interest was focused on explaining the independent effects of each covariate, all potential variables were included in the unadjusted model and adjusted model. This analysis was not focused on prediction [23]. The *p*-values, odds ratios (ORs), and 95% confidence intervals (95% CIs) were estimated in each analysis. The reliability estimate was employed to determine the final multilevel model’s adequacy. All variables theoretically associated with the outcome were kept in the final model.

## 3. Results

Teleconsulting sessions avoided patient referral to other care levels in 65.1% of the cases. Most PHC professionals that requested responses were female (65.3%) and from the Family Health Strategy (FHS) specialty (47.8%), followed by general dental practitioners (43.8%). Semiology/pharmacology/stomatology was the dentistry field with significant demand (45.7%), followed by general dentistry (40.6%) (Table 2).

Contextual variables were descriptively analyzed (Table 3).

The null model showed differences in the frequency of avoiding referrals of patients to other care levels by the teleconsulting sessions among the 279 municipalities assessed in this study (*p* = 0.000) (Table 4). In the final model, the contextual variables explained 44.23% of the variance in avoided patient referrals to other care levels.

The final adjusted multilevel analysis (n = 1319) indicated that female dentists were more likely to avoid patient referrals than male dentists (OR = 1.74; CI = 0.99–3.44; *p* = 0.055). An increase of one percentage point in OHT/PHC coverage of municipalities increased the likelihood of avoiding patient referral by 1% (OR = 1.01; CI = 1.00–1.02; *p* = 0.020) (Table 5).

## 4. Discussion

This study is original since it nationally assessed potentially associated factors with avoiding referrals by Brazilian dental teleconsulting. It might contribute to achieving a more resolute program and PHC, reducing waiting times to other care levels. The data period selected marks the onset of the COVID-19 pandemic, responsible for creating a new obstacle to accessing health services due to social distancing measures, which generated a repressed demand for treatments in the population [24].

The asynchronous teleconsulting sessions have reportedly been one of the most offered and used [25,26], despite the low rates of utilization of teledentistry services [15,16] within the modalities offered by the telehealth program to support professionals. In this study, most of the teleconsulting sessions requested in 2020 avoided referring patients to other care levels. It reinforces the potential of dental teleconsulting in contributing to PHC effectiveness [1,4,14,15,16,27]. Teleconsultants clarify PHC professionals’ concerns and confirm patients’ diagnoses, qualifying them for the referral process [13,26], saving resources, time, and stress [28].

As observed in previous studies, female dentists demanded teleconsulting sessions the most [29,30]. Moreover, women were 1.74 more likely to avoid patient referrals than men. Females are more likely to use the internet for assessing health-related services and information [31] and lead a healthy lifestyle, seeking more health care assistance [32,33]. Women tend to be more cautious concerning health care, which could influence their attitudes during teleconsulting sessions.

The significant requests for teleconsulting from FHS dental surgeons and general dental practitioners were expected due to the Brazilian PHC profile [34], and most questions were related to semiology/pharmacology/stomatology, followed by general dentistry, reflecting the conditions found constantly in PHC attendances. The potential for teleconsulting sessions should be explored more in the national context of the disparities and difficulties in the relationship between primary and secondary care, with long waiting lists for specialties such as endodontics, oral surgery, and periodontics [3] (included in general dentistry). Furthermore, the high demand for stomatology questions has been previously reported [16,29,30], followed by the difficulty in diagnosing oral diseases in PHC [13,16,29], the low rate of procedures for the early detection of oral cancer in OHTs, and many municipalities without reference services for stomatology cases [35].

The null hypothesis was rejected since the null model indicated a difference in the prevalence of avoiding referrals of patients to other care levels by the teleconsulting sessions in the municipalities assessed. The contextual variables partly explained the outcome variance. Contextual factors, such as the organization of local health systems and services, the size of municipalities, institutional support, and the program’s implantation, can influence telehealth utilization by PHC professionals. The heterogeneous incorporation of the telehealth program into the Brazilian territory, which presents marked developmental differences, must be considered to improve the program’s effectiveness and efficiency [26].

One percentage point increase in OHT/PHC coverage of municipalities increased the likelihood of avoiding patient referral by 1%. Municipalities with better OHT/PHC coverage are expected to have a more organized PHC network, which could reflect better potential patient access and capacity to solve problems. Including the OHT in the FHS restructured the PHC model. It reordered health action planning in the public sector, focusing on the territorial and epidemiological needs of the population [33].

Noteworthy is that 75% of the municipalities that requested teleconsulting showed rates of DSC/1,000,000 inhabitants of 0.00. In comparison, most of them had reasonable OHT/PHC coverage rates: the median OHT/PHC coverage was 76.52%, and P75% of the municipalities that requested teleconsulting showed an OHT/PHC coverage of 100%. However, 25% of the municipalities (P25%) still showed OHT/PHC coverage under 41.55%. This rate reinforces the need for a resolutive PHC in the country [2]. In the context of significant variability in healthcare coverage [3,26,33,35], performance [36], and infrastructure [4], telehealth is an essential strategy to narrow the gaps within care levels and reduce health inequalities [24,26].

Continuing professional development is related to better OHT performances, resulting in better patient care [37]. In this sphere, telehealth strategies must be stimulated since it provides access to several continuing professional activities, such as teleconsulting, contributing to increasing PHC resolution capacity.

Using secondary data, which hindered a richer collection of information regarding whether the avoided referral was unnecessary, and the loss of data due to the lack of PHC professionals’ feedback on the platform were some limitations of this study. The period analyzed was atypical because of the COVID-19 pandemic, with new challenges in the daily practice of health care services. Thus, future studies are needed to explore these issues further and direct interventions to improve the telehealth program’s results.

## 5. Conclusions

Teleconsulting sessions avoided most patient referrals to other care levels. The contextual variables partly explained the differences in the prevalence of avoided referrals. Female dentists and municipalities with better OHT/PHC coverage were more likely to avoid patient referrals to other care levels by teleconsulting sessions. We should focus on strategies to increase the equity in OHT/PHC coverage in Brazilian municipalities, better support both professionals and patients, and enhance the telehealth program’s resoluteness.

## Figures and Tables

**Table 1:** Description of each independent variable by level of analysis from dental teleconsulting sessions.

Variable	Description
Level 1—Individual ^1^	
Sex	Male–female
Dental specialty	FHS ^a^
	General dental practitioner
	Specialist
Dentistry field	General dentistry ^b^
	Semiology ^c^/pharmacology ^d^/oral medicine ^e^
	Services ^f^
	Health promotion and prevention ^g^
Level 2—Contextual ^2^	
mHDI	Municipal Human Development Index (0–1)
OHT ^h^ in PHC ^i^ coverage	Annual mean percentage (%) of the population covered by (OHT)/PHC
DSC ^j^ coverage	Rate of DSC/1,000,000 inhabitants
Illiteracy rate	Illiteracy rate of each municipality
Gini index	Gini index (0–1)
Longevity	Life expectancy at birth
Per capita income	Per capita income of each municipality

^1^ Data extracted from each teleconsulting session: dentist and teleconsulting-related factors. ^a^ Family Health Strategy; ^b^ dentistry, endodontics, and periodontics specialties; ^c^ diagnosis and systemic diseases; ^d^ prescription of medications and their adverse effects; ^e^ oral lesions; ^f^ patient referral, the health system, service operation, and administrative processes; ^g^ oral health instruction and preventive habits and practices. ^2^ Data from each municipality that requested teleconsulting sessions. ^h^ Oral health teams; ^i^ primary health care; ^j^ dental specialty centers.

**Table 2:** Descriptive analysis of individual variables from dental teleconsulting sessions.

Variable	N	%
Dependent variable		
Teleconsulting sessions avoided patient referral to other care levels		
Yes	859	65.1
No	460	34.9
Independent variables (individual level)		
Sex		
Female	861	65.3
Male	458	34.7
Dentist specialist		
FHS	631	47.8
General dental practitioner	577	43.8
Specialist	111	8.4
Dentistry field		
Semiology/pharmacology/stomatology	603	45.7
General dentistry	535	40.6
Services	96	7.3
Health promotion and prevention	85	6.4

**Table 3:** Descriptive analysis of contextual variables from municipalities (n = 279) that requested teleconsulting sessions.

Variable *	*p*-Value	P25%	P50%	P75%
mHDI	0.000	0.602	0.675	0.721
OHT/PHC coverage	0.000	41.550	76.520	100.000
DSC	0.000	0.000	0.000	0.000
Illiteracy rate	0.000	7.900	12.300	21.900
Gini index	0.012	0.450	0.500	0.540
Longevity	0.001	71.420	73.670	75.010
Per capita income	0.000	290.990	493.590	674.120

* Values for 279 municipalities; *p*-value = Kolmogorov–Smirnov normality test; P, percentile.

**Table 4:** Final estimation of variance components in the multilevel analysis (“null model”).

Random Effect	Standard Deviation	Variance Component	df	Chi-Square	*p*-Value
Intercept, U0	1.61533	2.60929	277	655.89	0.000 *

* *p* < 0.05.

**Table 5:** Multilevel models (unadjusted and adjusted) for variables of individual (n = 1,319) and contextual levels associated with avoiding patient referral to other care levels.

Variable	Avoided Referral (%)	Unadjusted OR *	95% CI	*p*-Value	Adjusted OR *	95% CI	*p*-Value
Individual level							
Sex							
Male	51.1	1			1		
Female	72.6	1.74	0.95–3.18	0.073	1.84	0.99–3.44	0.055
Dentist specialist							
FHS	68.6	1			1		
General dental practitioner	61.0	1.05	0.57–1.94	0.870	0.98	0.53–1.82	0.949
Specialist	66.7	1.13	0.41–3.09	0.813	1.18	0.43–3.29	0.745
Dentistry field							
General dentistry	73.8	1			1		
Semiology/pharmacology/stomatology	30.0	0.82	0.50–1.32	0.407	0.78	0.48–1.27	0.318
Services	56.2	0.77	0.36–1.64	0.496	0.74	0.34–1.60	0.445
Health promotion and prevention	40.0	0.85	0.34–2.13	0.722	0.78	0.31–1.98	0.606
Contextual level							
mHDI		6.78	0.14–333.33	0.332	0.000258	0.00–0.04	0.159
OHT/PHC coverage		1.01	1.00–1.02	0.016	1.01	1.00–1.02	0.020
DSC coverage		0.99	0.98–1.01	0.424	0.99	0.98–1.01	0.349
Illiteracy rate		0.99	0.96–1.02	0.524	1.00	0.96–1.03	0.928
Gini index		0.09	0.00–5.68	0.257	0.42	0.00–58.8	0.729
Longevity		1.03	0.92–1.16	0.550	0.96	0.78–1.18	0.710
Per capita		1.00	0.99–1.00	0.816	1.00	0.99–1.00	0.197

* OR = Odds Ratio.

## Data Availability

The data presented in this study are available on request from the author.
